# Influence of macronutrients and micronutrients on maize hybrids for biogas production

**DOI:** 10.1007/s11356-023-27235-3

**Published:** 2023-05-05

**Authors:** Mykola Grabovskyi, Petro Kucheruk, Kostantin Pavlichenko, Hynek Roubík

**Affiliations:** 1 Bila Tserkva National Agrarian University, 8/1 Soborna Sq, Bila Tserkva, Kiev, 09117 Ukraine; 2 Institute of Engineering Thermophysics, National Academy of Sciences of Ukraine, 2a, Marii Kapnist Str, Kiev, 03057 Ukraine; 3 Department of Sustainable Technologies, Faculty of Tropical AgriSciences, Czech University of Life Sciences Prague, Kamýcka 129, Suchdol, Prague, 16500 Czech Republic

**Keywords:** Silage, Crop yield, Dry matter, Biomethane, Economic efficiency, Energy efficiency

## Abstract

Important in the cultivation of corn for biogas production is the selection of appropriate hybrids, macro- and micronutrient dozing and the evaluation of energy and economic efficiency of their use. Therefore, this article presents the results of 3-year field research (2019–2021) on the yield of maize hybrids of different maturity groups grown for silage. The influence of the application of macronutrients and micronutrients on fresh and dry mass yield, chemical composition, methane yield, energy, and economic efficiency was analysed. It was established that depending on the maize hybrid, the application of macro- and micro-fertilizers increased the yield of the fresh mass of maize by 1.4–24.0% compared to options without their use. The evaluation of the theoretical yield of CH_4_ based on the content of fats, protein, cellulose, and hemicellulose is also presented in different samples of maize. The findings show that the application of macro-and micro-fertilizers is suitable from the energy and economic points of view — profitability begins to appear at the price of biomethane of 0.3–0.4 euros per 1 m^3^.

**Supplementary Information:**

The online version contains supplementary material available at 10.1007/s11356-023-27235-3.

## Introduction

Maize silage has been and remains one of the most common raw materials for biogas production in many EU countries and Ukraine. According to estimates from the Bioenergy Association of Ukraine, in 2019, 13 of the 26 biogas plants in Ukraine used maize silage. Its contribution to total biogas production is estimated at almost 50% (Geletukha et al. 2022). A Ministry of the Environment Health and Consumer Protection State of Brandenburg study (MUGV 2010) indicates that approximately 28% of maize silage in Germany was used for biogas production.

Maize is a popular agricultural crop that is widely grown in many countries (Graß et al. 2013). It has high fresh biomass and dry matter yields per hectare, high suitability for ensiling, and high biogas yield (Brauer-Siebrecht et al. 2016). Compared to other substrates, maize silage provides a more stable production of biogas and methane, which, as a result, facilitates the dosing of the substrate in the fermentation chamber and stabilises the operation of the biogas unit (Fugol and Szlachta 2010).

The basic indicator that affects the economic efficiency of the use of maize silage for biogas production is the methane yield per 1 ha of land. In turn, this indicator is a function of the interaction between the dry matter yield of maize (tDM/ha) and the specific methane yield (Nm^3^CH_4_/tDM).

Applying macronutrients and micronutrients, in general, has a positive effect on the yield of maize. Nitrogen (N) application increases the dry matter yield of maize silage, increases the protein ratio, and decreases the acid detergent fiber (ADF) and neutral detergent fiber (NDF) ratio in silage samples (Uzun et al. 2020). In the boreal regions, the N application rate of 100–150 N kg ha^−1^ for forage maize is recommended. There is no need to increase the application of N, as climate conditions seem to limit the growth and N recovery efficiency (N_RE_) of forage maize (Liimatainen et al. 2022).

A raised level of N fertilization from 100 to 300 kg ha^−1^ increased plant height, plant diameter, green herbage yield, crude protein, metabolic energy, gas production and organic matter digestibility, and decreased pH levels, ADF and NDF ratios (Kaplan et al. 2016).

The results (Oleszek and Matyka 2018) showed that a raised N from 40 to 160 kg ha^−1^ increased biogas and methane yield, and the specific yield for the six energy crops, namely maize, sorghum, sunflower, triticale, reed canary grass, and Virginia mallow. The highest increase in methane yield was observed in Virginia mallow from 145 to 197 dm^3^ kg^−1^ of volatile solids (VS) due to biodegradability increase by 15%.

Uzun et al. (2020) concluded that the yield of maize and its quality improve significantly with the application of 7.5 kg N da^−1^ as a starter fertilizer at the sowing stage and 15.0–22.5 kg N da^−1^ as top dressing in the 6-leaf stage.

Amanullah Kakar et al. (2014) noted decreased maize grain yield with the sole application of N, P and K in a split (2% each) in the 30 days after emergence (DAE). At the same time, the combined application of N + P and N + P + K in a split at 30 DAE or 60 DAE or in two equal splits (1% each at 30 and 60 DAE) increased the yield of the maize grain. Furthermore, the results showed the increased productivity of maize with the combined application of the N + P + K at a rate of 1% each in two equal splits at 30 and 60 DAE.

The grain yield of maize increased by 43% with the application of half N as base and half N as foliar spray compared to that obtained by applying full N (100 kg N/ha) (Islam et al. 1996). Foliar fertilization with nutrients cannot replace soil fertilization in the case of maize and it is recommended to use it as a supplement to fertilizers applied to the soil-applied fertilizers (Ling and Silberbush 2002).

Until recently, the use of micronutrients on crops was considered as an additional and optional technological measure. However, in some studies, the importance of all nutrients in shaping plant productivity was shown (Safdarian et al. 2014; de Campos Bernardi et al. 2011; Karlen et al. 1985). Therefore, both macronutrients and micronutrients play the key role in the fertilisation of crops, including maize.

Maize is susceptible to low Zn in soils (Alloway 2008). A very positive response of maize plants to increasing soil Zn application was pronounced in the case of ZnSO_4_ application (Grujcic et al. 2021). The use of concentrated stilbite (650 g) or natural zeolite (470 g) with urea increases the dry matter yield of maize for silage and the concentration of nitrogen in the leaves (de Campos Bernardi et al. 2011).

The use of micronutrients and growth regulators in maize crops has a positive effect on plant growth and, in turn, on crop formation. Regardless of the maturity group of hybrids, the application of micronutrients increases the grain yield of the maize hybrids by 0.38–1.26 t/ha by 3.8–10.0% (Lavrynenko et al. 2016).

Amon et al. (2007) revealed the regression dependence of the specific methane yield of maize silage on the content of crude protein, crude fat, cellulose, and hemicellulose. The maturity phase of maize significantly affects the content of dry matter in the silage and the ratio of individual groups of organic compounds in its composition. The methane yield per hectare of late ripening maize varieties in different stages of vegetation is estimated to be in the range of 6000 to 9000 Nm^3^CH_4_/ha (Amon et al. 2007). The specific methane yield decreased towards full ripeness, from 312–365 NlCH_4_/kgVS (milk ripeness) to 268–286 NlCH_4_/kgVS (full ripeness) (Amon et al. 2007).

Szempliński and Dubis (2011) noted the dry matter yield (DM) of 23.8 t ha^−1^ and energy intensity of maize cultivation of 21.7 GJ ha^−1^ with fertilization of 180 kg N ha^−1^, while Barbanti et al. (2014) reported the energy input for cultivation of sorghum and perennial grasses at applying 120 kg N ha^−1^, amounted to 21 and 15 GJ ha^−1^, respectively. At the same time, the energy use efficiency (EUE) of biogas production from maize was much lower than that of sorghum and grass species due to the significant differences in the energy demand for the crops cultivation. Gerin et al. (2008) determined that EUE of biogas production from grass ranged from 7 to 14 GJ GJ^−1^ and for maize — from 7 to 25 GJ GJ^−1^.

Oleszek and Matyka (2020) found that an increase in the N dose from 40 to 160 kgN ha^−1^ significantly increased biomass yield and methane output, while causing an increase in energy input. However, the application of higher doses of N did not cause an extreme decrease in energy use efficiency. The energy use efficiency for harvested in two cuts sorgum (5.0–5.2 GJ GJ^−1^) was close to the energy use efficiency of maize (5.7–6.8 GJ GJ^−1^), in spite of the much lower methane productivity (2027–2903 m^3^ ha^−1^ and 4409–5692 m^3^ ha^−1^, respectively) and the energy output (73–105 GJ ha^−1^ and 159–205 GJ ha^−1^, respectively).

Szempliński et al. (2014) noted that high level of nitrogen fertilization significantly increases the biomass yield, but at the same time, significantly decreases the energy use efficiency. The importance of the species was also emphasized. Much less energy consumption was observed when cultivating multiannual Virginia mallow species compared to maize and sorghum (16.2; 21.3 and 18.3 GJ ha^−1^, respectively). However, due to the differences in biomass yield, the energy use efficiency was still the highest for maize.

Barbanti et al. (2014) noted that despite the high yields of biomass and methane, low energy use efficiency of biogas production from maize is observed due to high energy costs for its cultivation. Meanwhile, other tested crops, such as sorghum, Arundo, and switchgrass, deserve attention, given the low energy need for their cultivation. At the same time, these crops have lower biodegradability and methane yields compared to maize silage.

Currently, there are not enough studies on the impact of fertilizers on energy and economic efficiency of biogas production from energy crops, including maize. In addition, most of these studies reduce the role of fertilisation level to a helpful effect on biomass yield. Meanwhile, it was found that differentiated fertilization rates affect not only biomass yield, but also biomass chemical composition and biodegradability, and thus biogas production efficiency (Kacprzak et al. 2012). Oleszek and Matyka (2020) proved that increasing the level of nitrogen fertilization increases biogas yield due to positive changes in the chemical composition of biomass, mainly due to a decrease in lignin content and improved digestibility.

Therefore, this study aims to assess the influence of macro- and micronutrients on the quality indicators of maize hybrids, their productivity, methane yield, and the economic and energy efficiency of their cultivation.

## Materials and methods

### Field experiment

For cultivation and further analysis, 4 different maize hybrids of the company KWS SAAT SE & Co, Germany were selected, namely: the mid-early hybrid Amaros (FAO 230), the mid-early hybrid Bogatyr (FAO 290), the mid-ripening KWS 381 (FAO 350) and the mid-late hybrid Carifols (FAO 380). During 2019–2021, a 3-factor field experiment was conducted with 4 maize hybrids and macro fertilizers (NPK), as well as additional seed treatment and spraying of plants with micro fertilizers. For each type of maize hybrid, 9 different combinations of exposure factors were applied, as shown in Table 1.

**Table 1:**
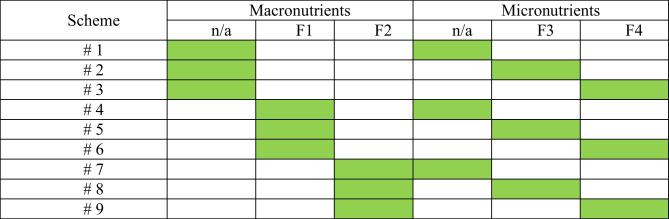
Staging an experiment*

* — the green cells indicate the different combinations of exposure factors applied in each scheme; **n/a**, not applied; **F1** — N_90_P_60_K_60_; **F2** — N_120_P_90_K_90_; **F3** — maize seeds treated with micronutrient *YaraVita Teprosyn NP* + *Zn* (liquid fertilizer with nitrogen content of 9%, phosphorus — 6.6%, zinc — 18%; dozing 5 L per ton) followed by spraying plants at 3–5 leaves stage with micronutrient *YaraVita Maize Boost* (a liquid fertilizer containing phosphorus (29.5%), potassium (5.0%), magnesium (4.5%) and zinc (3.1%); dozing 4 L per 1 hectare); **F4** — maize seeds treated with micronutrient *YaraTera Tenso Cocktail* (stable water-soluble mixture of iron (2.1%), manganese (2.57%), copper (0.53%), calcium (2.57%), boron (0.52%), zinc (0.53%) in chelated form (EDTA); dozing 0.15 kg per ton) followed by spraying plants at 3–5 leaves stage with micronutrient *YaraVita Kombiphos* (a liquid fertilizer with phosphorus content (29.7%), potassium (5.1%), magnesium (2.7%), zinc (0.3%) and manganese (0.7%); dozing 3 L per 1 ha). Winter wheat was the previous crop in each year of the experiment

In four replicates, the total area was 224 m^2^, the accounting area — 186 m^2^, in a randomised complete block design in a ‘split plot’ system.

The macro fertilizer was used as complex fertilizer under the trademark “nitroamophoska” (N_18_P_18_K_18_), applied in the fall for ploughing. Nitrogen fertilisation in the form of ammonium nitrate (N_33_) was applied before maize sowing.

### Soil and climate characteristics of the research site

The field experiments were carried out at the “Korobivsky poultry farm”, located in the Andrushiv district of the Zhytomyr region of Ukraine. Soil — chernozem podzolic medium clay. The pH of the soil was 6.7, and the concentrations of nutrients in the soil were determined in the following range: organic carbon (C_org_) — 2.54%, nitrogen — 87.50 mg kg^−1^, phosphorus — 117,9 mg kg^−1^, potassium — 110.1–140.5 mg kg^−1^. The organic carbon content in the soil was determined using the method Walinga et al. (2008). A digital pH meter (AMT-300, China) was used to measure the soil pH. The mobile forms of P and K were extracted by the Egner et al. method (1960).

The data of the meteorological station in Zhytomyr, located 42 km from the research sites, was used to characterise the weather conditions. Table 2 shows the average rainfall (SoP) and temperature (T) for the maize-growing season in 2019–2021.

**Table 2:** Precipitation and temperature at the research site

Period	2019		2020		2021		Average 2019–2021	
	SoP, mm	Mean T, °C	SoP, mm	Mean T, °C	SoP, mm	Mean T, °C	SoP, mm	Mean T, °C
April	32.7	10.3	38.7	9.1	44.6	7.7	42.0	6.6
May	67.5	15.6	128.3	12.0	115.7	13.7	58.0	13.6
June	42.6	22.4	79.8	20.3	53.6	20.3	74.0	16.3
July	38.7	18.8	46.5	20.3	42.4	24.3	85.0	18.4
August	12.3	20.9	39.4	20.6	44.3	21.3	52.0	17.3
Over April–August	193.8	17.6	332.7	16.5	300.6	17.5	311.0	14.4

The experimental area has a temperate continental climate with cold winter and hot and dry summer. The average long-term temperature of the coldest month (January) is − 6 °C, the warmest (July) is + 17–19 °C. The average annual temperature in the region is + 6…7 °C. The growing season (days with an average air temperature above 5 °C) lasts from the second decade of April to the third decade of October. The weather was warmer than average during the experimental periods in all years. While April and May were warmer in the first experimental year, June, July, and August were warmer in all years compared to the average. Although May received more precipitation than average in 2019 (Table 2), it was not sufficient to meet the water needs of the maize plant for the entire growing season. In 2020 and 2021, there was a sufficient amount of precipitation, and in May, even in excess, to form a high yield of green mass of maize. In general, except for the first experimental year, the precipitation received during the growing season 2020–2021 was sufficient for the formation of a high yield of maize for silage.

### Crop yield

The yield of the green mass was determined by weighing plants from the accounting plots, followed by recalculation per hectare. The different hybrids of maize plants were harvested at the beginning of the reproductive phases: R3 (pasty grain), R4 (floury grain) and R5 (hard grain). The grains in the R3 stage had a yellow exterior color with a milky to pasty internal fluid. At the beginning of stage R4, the milky internal fluid becomes thicker, reaching a pasty consistency. In stage R5, all or almost all grains are floury-hard, according to Ritchie et al. (2003). Twenty plants were randomly chosen from each plot and cut to the ground level to determine biological yield in phases R3 and R4. In phase R5, harvesting was performed for the entire area of each plot after taking out one row from each side of the plot and 45 cm from the beginning and the end of each row.

**Fig. 1:**
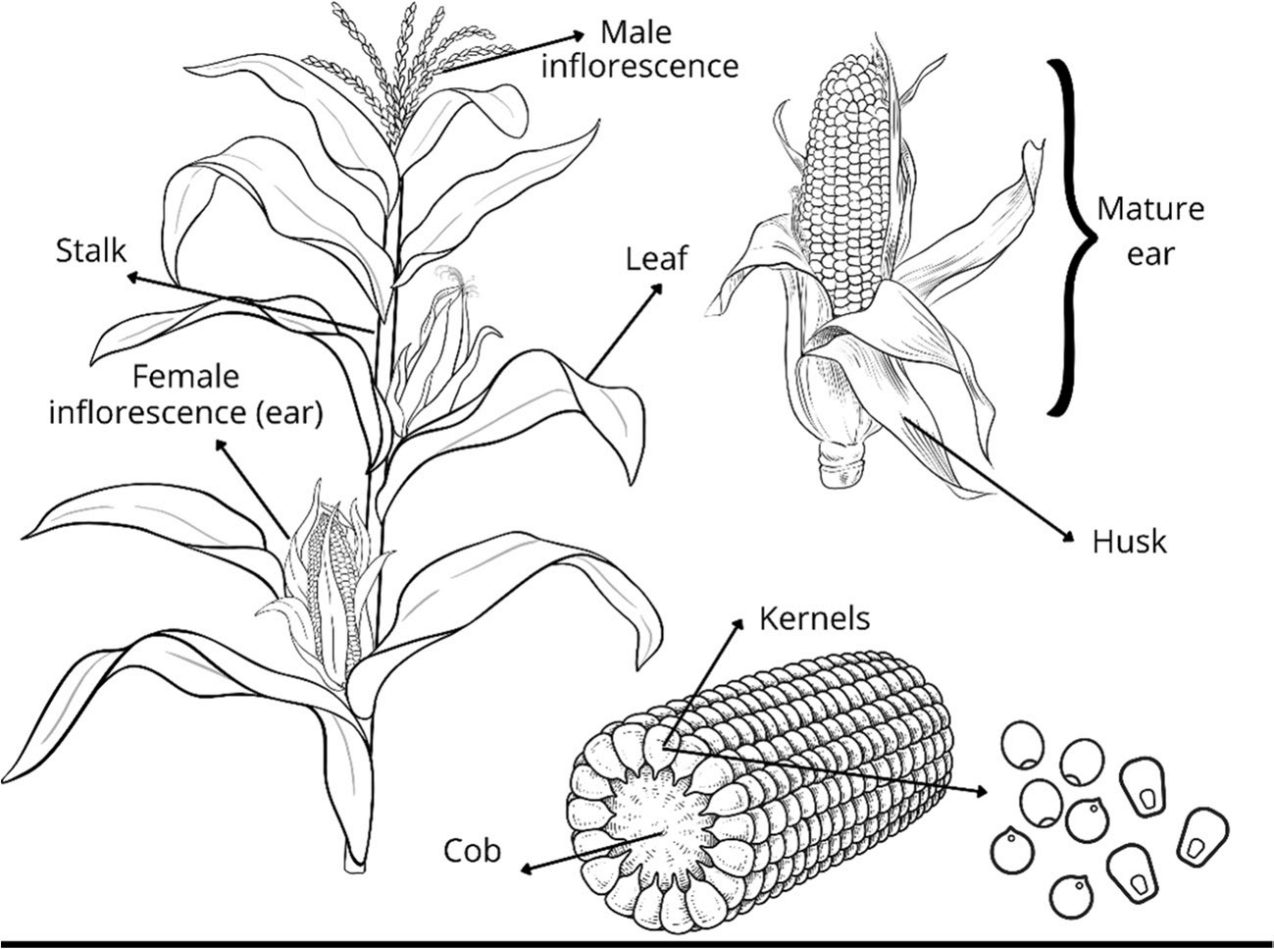
Structural parts of the maize plant

Before each phase (R3, R4, R5), two whole plants from each plot were randomly selected and partitioned into five fractions: leaf, stalk and the mature ear structure (grain, cob, husk) (Fig. 1). The cob structure consisted of the portion of the cob without grain plus the husk. These fractions were dried individually to determine their DM content. The proportion of these fractions was then calculated on a DM basis. The dry weight of each plant fraction was determined as above for the whole-plant sample.

### Chemical characteristics

The chemical analysis of the maize plant samples was carried out at the Eurofins Agro laboratory (BLGG AgroXpertus) in Kyiv. The fresh weight of the 5 maize plant samples was determined at the beginning of the reproductive phases. The plants were then chopped into 2.5–3.0 cm pieces, filled and sealed in 2 kg deflated vacuum bags to provide material to determine the dry matter and chemical analyses. The raw material was combined, fragmented, ensiled in 5-L plastic barrels, and stored in the dark. The dry matter (DM) was determined using a gravimetric method after drying at 105 °C (Oleszek and Krzemińska 2017).

The crude protein (CP) content was determined using the Kjeldahl method (Bremner and Breintenbeck 2008). Ash concentration was determined in a muffle furnace at 550 °C for 6 h. The starch was determined using the procedure of Holm et al. (1986). The fat content of the samples was determined by continuous solvent extraction using a Soxhlet apparatus according to the methods of Hughes (Hughes 1969). The crude fiber was determined combined the Crude Fiber methods for Maize (A-8) and Feedstuffs (G-12) (Official Methods of Analysis 1984). The contents of hemicellulose, cellulose, and lignin were analyzed using the Van Soest method (Van Soest 1963).

### Methane yield

The methane energy values (MEV) of the maize hybrids were estimated with the use of the multiple linear regression equation proposed by Amon T. et al. (2007), as follows Eq. (1):

1 $$\mathrm{MEV}=19.05\times \mathrm{CPr}+27.73\times \mathrm{CFt}+1.8\times \mathrm{Cel}+1.7\times \mathrm{HCel}$$

CPr: crude protein content (% in TS).
CFt: crude fat content (% in TS).
Cel: cellulose content (% in TS).
HCel: hemicellulose content (% in TS).

Biogas productivity (BP) (2) and methane productivity (MP) (3) per hectare were estimated using biogas yield (BY) and methane yield (MY) according to the following equation (Oleszek and Matyka 2018):

2 $$\mathrm{BP}=\mathrm{Y}\times \mathrm{BY}$$

3 $$\mathrm{MP}=\mathrm{Y}\times \mathrm{MY}$$

where BP is the biogas productivity (m^3^ ha^−1^), BY is the biogas yield (m^3^ t^−1^), MP is the methane productivity (m^3^ ha^−1^), MY is the methane yield (m^3^ t^−1^), and Y is the silage yield (t ha^−1^).

### Statistical analysis

Statistical analysis was performed using Statistica 12.0 software (StatSoft) to explain the influence of maize hybrids, macronutrients and micronutrients, and year of cultivation on biomass yield and methane productivity. Hybrids were considered fixed effects and replications random effects. When *F*-ratios were significant (*P* < 0.05), LSD values at that level were used to compare treatment means.

### “Net” profit and “net” energy surplus estimation (energy output and input)

To assess the influence of macronutrient and micronutrient application factors on the economic feasibility of using silage for biogas production, the costs of growing maize hybrids, including the full cycle of growing, harvesting and grinding “in the field”, were estimated. The energy consumption estimation also considers fuel consumption (diesel) in the full cycle of growing, harvesting and grinding maize “in the field”. Estimates were made for the R5 wax phase maize crop.

Conditionally, net profit is estimated as the difference between the potential income from the sale of biomethane from maize grown on 1 ha (in the price range of 200–1000 EUR/1000 m^3^) and the cost of silage production in the field based on 1 ha. The assessment of the possible income from the sale of biomethane does not consider all the costs of its production in the entire chain, from transportation from the field to the biogas station to the supply of biomethane to the natural gas network.

Similarly, the relative net energy surplus is estimated as the difference between the gross energy potential of biomethane from maize grown on 1 ha and the fuel energy used to produce maize silage from 1 ha.

The energy gained from the production of methane, the so-called energy output (E_o_), was calculated according to the following equation (Oleszek et al. 2016) Eq. (4):

4 $${E}_{o}=MP\times 35.8\times {10}^{-3}$$

where E_o_ is the energy output (GJ ha^−1^), MP is the methane productivity (m^3^ ha^−1^), and 35.8 is the methane lower heating value (MJ m^−3^).

The energy input (EI) calculation includes the energy demand for biomass cultivation and harvest. In the calculation of E_I,_ four main energy streams were defined: fuel (diesel), fixed assets (machines, tools, spare parts), raw materials (fertilizers, plant protection products, seeds and cuttings) and labour.

Energy use efficiency (EUE) was expressed as the ratio of E_o_ and E_i_ Eq. (5):

5 $$\mathrm{EUE}=\frac{{E}_{O}}{{\mathrm{E}}_{\mathrm{I}}}$$

where EUE is the coefficient of energy use efficiency (GJ GJ^−1^), E_o_ is the energy output (GJ ha^−1^), and E_I_ is the energy input (GJ ha^−1^).

The evaluation of the gross energy potential of biomethane does not consider all further energy consumption in the entire chain, from the field transportation to the biogas plant to the biomethane supply to the natural gas network.

## Results and discussion

### Fresh crop yield

The optimal time to harvest maize occurs when the product from the specific methane yield and the VS yield per hectare reaches maximum. In FAO 240–390 hybrids, this period occurs at the end of waxy grain maturity phase. Then maize has a dry matter content of 35–39% (Amon et al. 2004).

The fresh mass (FM) yield for the studied maize hybrids ranged from 22.8 to 57.5 t/ha (Table 3).

**Table 3:** The yield of fresh mass for the studied maize hybrids, t/ha

Hybrid	Macro-fertilizers	Micro-fertili-zer	The period of accounting								
			Kernel milk stage (R3)			Kernel dough stage (R4)			Kernel dent stage (R5)		
			2019	2020	2021	2019	2020	2021	2019	2020	2021
Amaros	-	-	23.6	41.2	37.1	24.7	43.7	39.1	22.8	41.1	36.4
		M1	24.4	42.0	38.5	25.5	44.5	40.6	23.6	41.8	37.8
		M2	24.8	42.2	38.6	25.9	44.6	40.7	24.0	42.0	37.9
	N_90_P_60_K_60_	-	27.2	45.8	43.2	28.4	48.7	45.5	26.3	45.8	42.4
		M1	28.0	46.4	43.9	29.3	49.1	46.3	27.1	46.1	43.1
		M2	28.5	46.5	44.0	29.8	49.2	46.4	27.6	46.3	43.2
	N_120_P_90_K_90_	-	29.6	48.2	45.4	30.9	51.3	47.9	28.6	48.2	44.6
		M1	30.3	49.0	46.2	31.7	52.1	48.7	29.3	49.0	45.4
		M2	30.8	49.2	46.4	32.2	52.2	48.9	29.8	49.0	45.6
Bohatyr	-	-	24.3	42.5	40.0	25.4	45.0	42.2	23.5	42.3	39.3
		M1	25.1	43.1	40.8	26.2	45.7	43.0	24.3	43.0	40.1
		M2	25.5	43.2	41.0	26.7	45.9	43.2	24.7	43.1	40.3
	N_90_P_60_K_60_	-	28.6	48.5	46.3	29.9	51.5	48.8	27.6	48.4	45.5
		M1	29.3	49.1	47.2	30.6	52.2	49.8	28.3	49.1	46.4
		M2	29.7	49.2	47.4	31.0	52.3	50.0	28.7	49.2	46.6
	N_120_P_90_K_90_	-	30.4	49.2	47.6	31.8	52.1	50.2	29.4	49.0	46.8
		M1	31.1	50.0	48.3	32.5	53.1	50.9	30.1	49.9	47.5
		M2	31.7	50.2	48.5	33.1	53.4	51.1	30.6	50.2	47.7
KWS 381	-	-	26.7	45.0	41.8	27.9	47.6	44.1	25.8	44.8	41.1
		M1	27.4	45.9	42.3	28.6	48.7	44.6	26.5	45.8	41.6
		M2	28.0	46.0	42.5	29.3	48.9	44.8	27.1	45.9	41.7
	N_90_P_60_K_60_	-	29.8	50.1	46.5	31.1	53.0	49.0	28.8	49.8	45.7
		M1	30.6	51.2	47.2	32.0	54.4	49.8	29.6	51.1	46.4
		M2	31.2	51.4	47.4	32.6	54.6	50.0	29.9	51.4	46.6
	N_120_P_90_K_90_	-	31.2	52.3	47.9	32.6	55.4	50.3	30.2	52.1	46.9
		M1	31.7	52.9	48.5	33.1	56.1	51.1	30.7	52.7	47.7
		M2	32.2	53.0	48.8	33.7	56.3	51.5	31.1	52.9	48.0
Karifols	-	-	27.9	42.4	44.2	29.2	44.8	46.5	27.0	42.1	43.4
		M1	28.5	42.9	44.7	29.8	45.5	47.1	27.6	42.8	43.9
		M2	29.0	43.0	44.8	30.3	45.7	47.2	28.0	42.9	44.0
	N_90_P_60_K_60_	-	30.2	51.4	48.3	31.5	54.4	50.9	29.2	51.2	47.5
		M1	30.6	52.0	48.9	32.0	55.2	51.6	29.6	51.9	48.1
		M2	30.9	52.2	49.1	32.3	55.5	51.8	29.9	52.2	48.2
	N_120_P_90_K_90_	-	32.4	53.1	50.7	33.9	56.2	53.5	31.3	52.8	49.8
		M1	32.8	53.9	51.4	34.3	57.1	54.2	31.7	53.7	50.5
		M2	33.3	54.1	51.5	34.8	57.5	54.3	32.2	54.0	50.6
LSD,* P* < 0.05			1.6	2.1	1.8	1.8	1.9	2.0	1.8	2.0	2.0

The most intensive growth of the green mass of maize hybrids occurred before the phase (R4), followed by a decrease of 5.2–6.8% in the phase (R5). Thus, in phase (R3), the yield of green mass of Amaros and Bohatyr hybrids was 34.0–43.5 t/ha, in phase (R4) – 35.8–45.9 t/ha, and in phase (R5), 33.4–42.8 t/ha. These indicators were 37.8–46.3, 39.9–48.9, and 37.2–42.6 t/ha for KWS 381 and Karifols hybrids, respectively.

On average, the Karifols maize hybrid provided the highest green mass yield in terms of fertilizers, while the Amaros hybrid provided the lowest. The difference between maize hybrid production, on average for 2019–2021 in the R3 phase, ranged from 3.1–6.2% to 10.0–12.3%, depending on the use of macronutrients and microfertilizers.

**Fig. 2:**
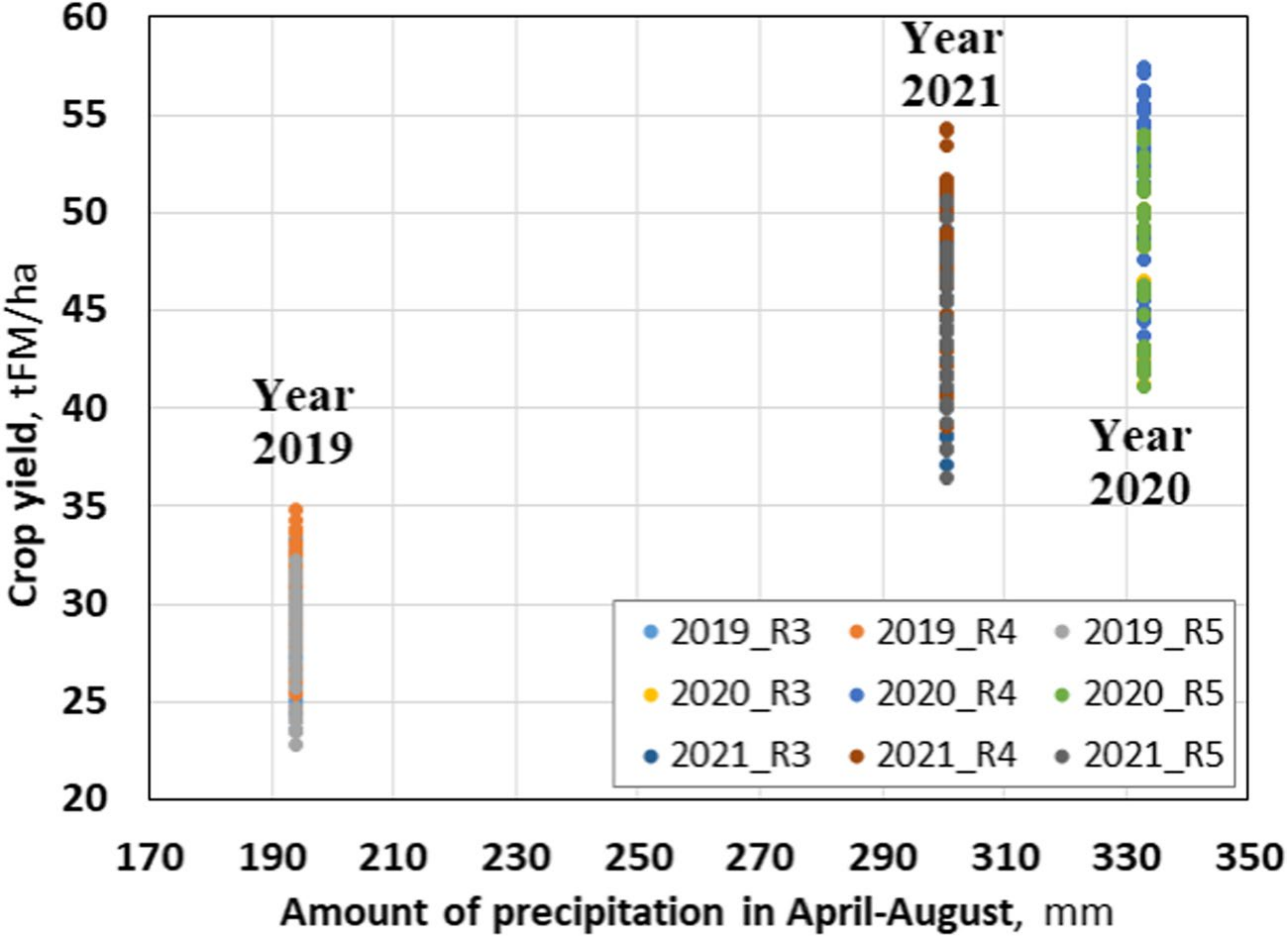
Maize crop yield vs the amount of precipitation (2019–2021)

The leading indicator that affected the yield of green mass was the amount of precipitation from April to August. In 2019, the amount of precipitation for April–August was 193.8 mm, and the average yield of green mass, according to the experiment, was 29.2 t/ha. In 2020, it was 48.9 t/ha (sum of precipitation 332.7 mm), and in 2021 — 46.0 t/ha (sum of precipitation 300.6 mm). From Fig. 2, the clear dependence of the yield of green mass on the amount of precipitation by year can be seen.

A better moisture supply in 2021 led to an increase in the yield of green mass of maize by 50.6–67.3%, and in 2020 by 50.6–80.1%, compared to 2019 (Supplementary information S.1). At the same time, most of the increase in yields is characteristic of the later stages of maturity of maize (R4 and R5), which is especially noticeable in the most the moisture-reliable year of 2020.

**Fig. 3:**
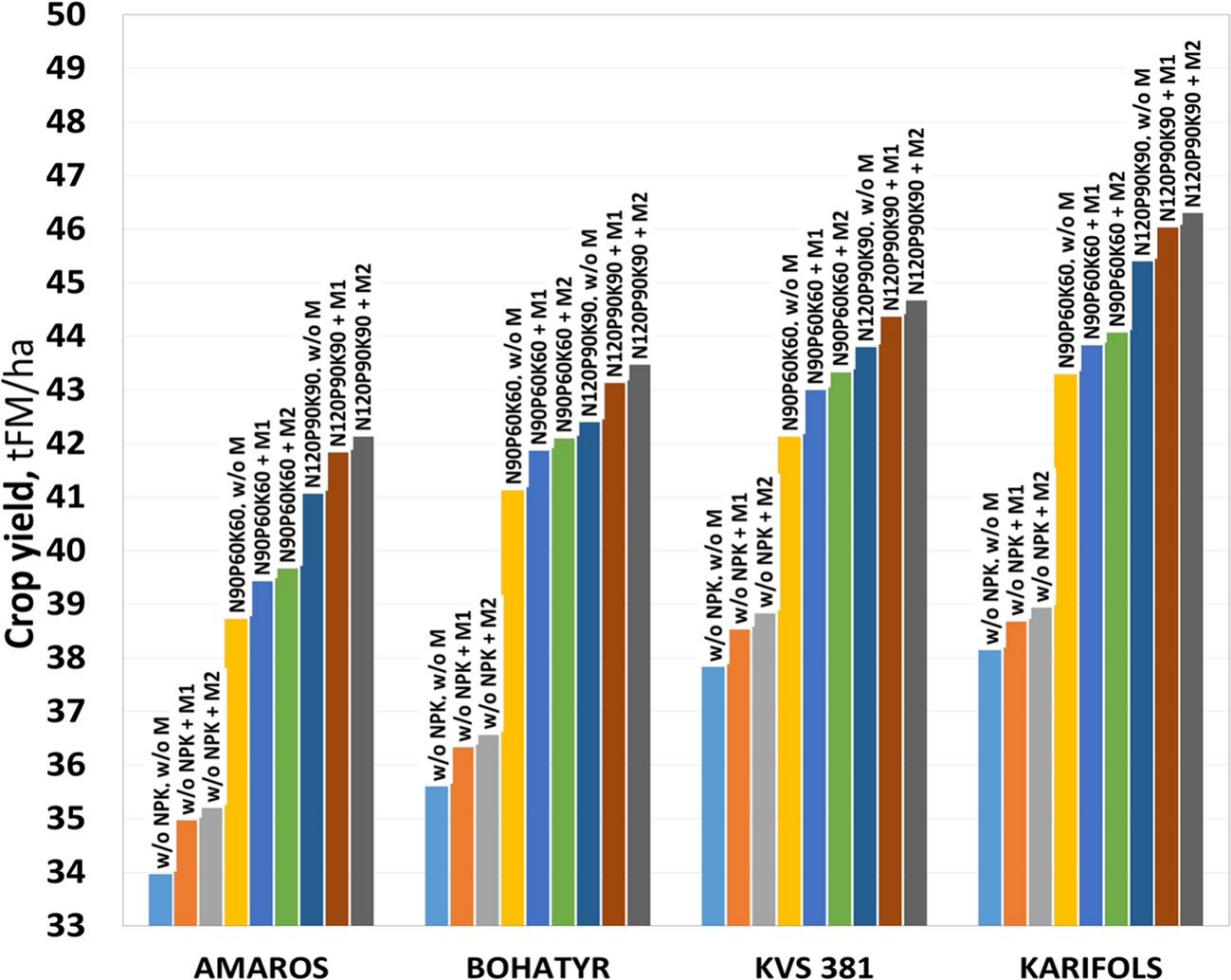
Maize crop yield (stage R3), average value for 2019–2021

The second most influential factor on maize yield was macronutrient application (Fig. 3).

In phase R4 on the variant without fertilizer application, the yield of fresh mass was 36.6, 38.1, 40.5, and 40.7 t/ha for hybrids Amaros, Bohatyr, KWS 381, and Karifols, respectively, while N_90_P_60_К_60_ application increased the FM yield on average by 11.4–15.5%, and N_120_P_90_К_90_ application — by 15.8–21.0%.

The use of micronutrients in the F3 scheme increased the yield of the green mass of maize by 1.4–2.9% compared to unfertilized options. When applying micronutrients according to the F4 scheme, the FM yield increased by 2.1–3.6%. It should be noted that there is no significant difference (LSD, *P* < 0.05) between F3 and F4 variants of micronutrient application in all studied hybrids.

Thus, the amount of precipitation during the growing season, the application and dose of macronutrients (NPK), and the maturity group of the maize hybrid (FAO) have the greatest effect on the maize FM yield and the least effect has the use of microfertilizers.

The highest FM yield of 48.9 t/ha was obtained for Carifols hybrid when N_120_P_90_К_90_ was applied, seeds were pretreated with YaraTera Tenso Cocktail 0.15 kg/t, and spraying of maize in the phase of 3–5 leaves with YaraVita Kombiphos 3 L/ha was applied.

### Dry matter yield

**Fig. 4:**
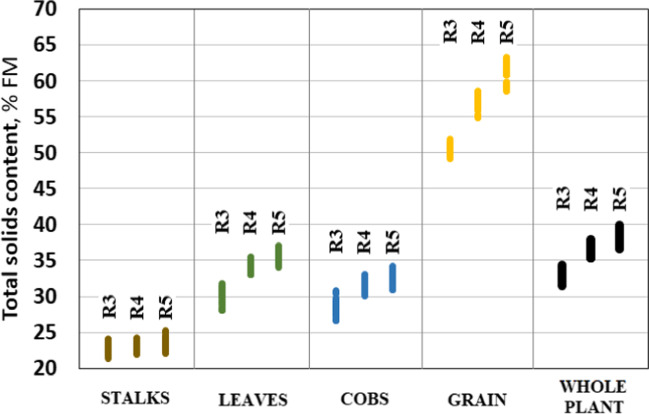
Average total dry matter content in different parts of maize plant

According to the experimental variants, the content of DM in the whole plant ranged from 31.5 to 39.9%. The average DM content ranged from 33.1% for phase R3 to 38.4% for phase R5. The dry matter content in individual parts of the maize plant differs significantly, as shown in Fig. 4.

The lowest DM content was in the stalks, on average 23.0–23.6%, and the highest in the grain — 50.6–61.3%. An increase in the FAO group of maize hybrids leads to an increase in the proportion of DM in different parts of the plant, to a greater extent in grain, leaves, and cobs + husks, and a lesser extent in stalks. Similar data were obtained by Schittenhelm (2008). They noticed that the fraction of vegetative plant parts (leaves and stalks) and the dry matter content in them increased considerably with the increasing maturity of the hybrid.

In phase R4, the DM content in the whole plant increased by an average of 10.5% (from 8.3 to 12.2%) compared to phase R3. In phase R5, the DM content was higher by 5% (from 3.4 to 6.6%) compared to phase R4.

According to Vildflush et al. (1995) application of mineral, fertilizers makes it possible to reduce water consumption for the formation of plant dry matter by 20–36%.

**Fig. 5:**
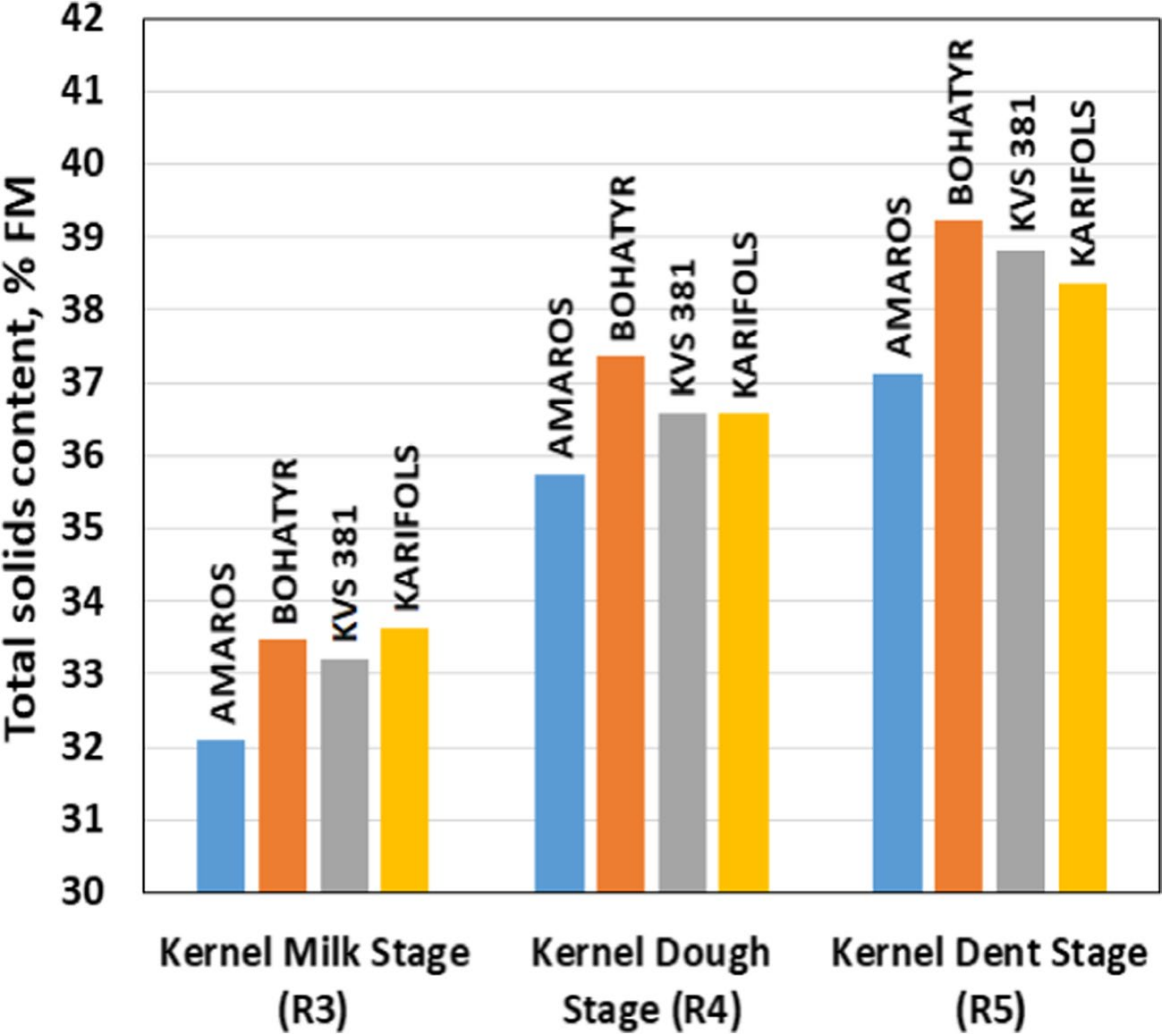
Total dry matter content in the studied maize hybrids at different maturity stages

No clear dependence of the effect of the addition and dosage of macronutrients and micronutrients on the DM content was found in the maize hybrids studied. In hybrids Amaros and Bogatyr, the content of DM in grain was 56.3 and 55.1%, in leaves — 33.5 and 32.5%, in stalks — 23.2 and 22.5%, in cobs + husks — 31.0 and 29.9%, and hybrids KWS 381 and Karifols — 56.3 and 56.5, 33.5 and 33.9, 23.1 and 23.3 and 33.5 and 33.9%, respectively. On average, the Bogatyr hybrid was marked with the highest DM content — 39.2%, and for the Amaros, KWS 381 and Karifols hybrids, this indicator was − 37.1, 38.8 and 38.4%, respectively (Fig. 5).

In phase R3, the Amaros and Bogatyr hybrids in the variant without fertilizer use had a DM yield of 11.1–12.4 tDM/ha, in phase R4 — 13.0–14.6 tDM/ha, and in phase R5 — 12.6–14.3 tDM/ha. Medium-ripe hybrids KWS 381 and Carifols yielded 12.7–13.2 tDM/ha, 14.8–15.2, 14.6–14.9 t/ha, in phases R3, R4, and R5, respectively (Supplementary information S.2).

With N_90_P_60_K_60_ application, DM yield increased, on average for hybrids, by 1.3–2.0 t/ha or by 11.4–15.5%, and with N_120_P_90_K_90_ application — by 1.7–2.9 t/ha or 12.8–17.0%, compared to options without their use. The effect of applying macronutrients on the yield of DM was higher than that of microfertilizers, as it was established for the fresh mass of maize.

According to the data received by Çarpici et al. (2010), increasing nitrogen rates increased the forage maize dry matter yield, with a peak value occurring at 300 and 400 kg N ha^−1^.

The use of micronutrients according to the F3 scheme provided an increase in the yield of DM by 1.2–2.9%, and according to the F4 scheme by 2.2–3.9%, compared to the options without their use. At the same time, no significant difference between micronutrient variants F3 and F4 was found.

The highest DM yield was obtained for the Carifols maize hybrid in the R4 phase in the F2 and F4 variants — 17.7 t/ha.

### Chemical composition

The key factors for the release of methane from energy crops and other substrates are the chemical composition of the substrate and its ability for biodegradation. The content of crude fat, crude protein, cellulose, hemicellulose, starch, crude fiber and sugars affect the formation of methane (Amon et al. 2007). There is a fairly significant number of models that predict the output of methane based on the chemical properties of the substrates. Lignin is the main inhibitor of methane formation (Thomsen et al. 2014). Dandikas et al. (2014) established a significant negative correlation between biogas and methane yields (*r* = 0.90) under acid detergent lignin content below 10% of total solids.

**Fig. 6:**
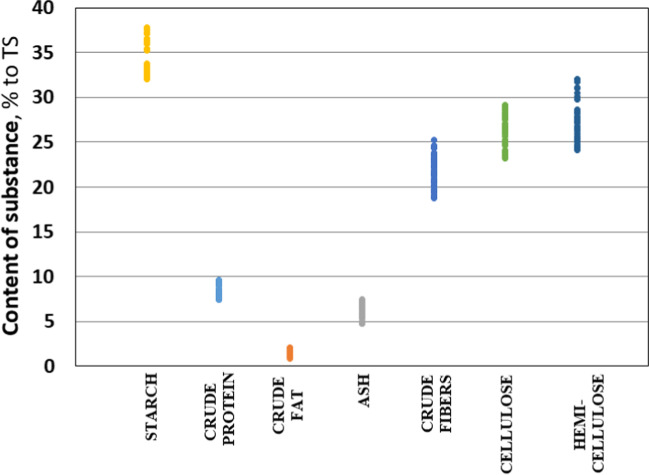
Chemical composition of dry matter of different maize hybrids

The crude protein content in the maize samples was 7.8–10.0% to DM, crude fat — 1.3–2.5%, cellulose — 23.6–29.6, hemicellulose — 24.5–32.4% (Fig. 6).

Tsavkelova and Netrusov (2012) established a negative correlation between biogas yield and ash and protein content in the studied samples.

**Fig. 7:**
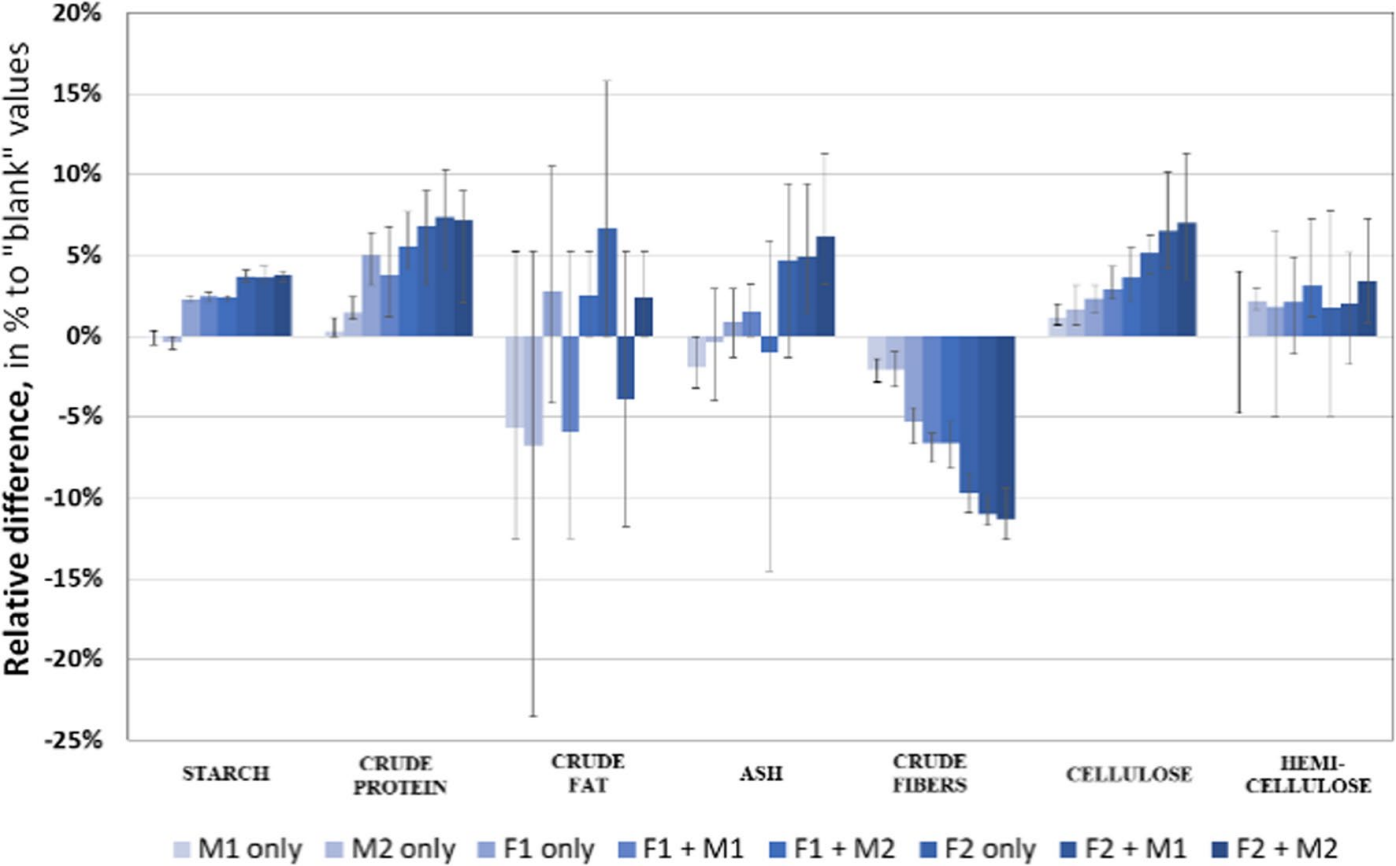
Relative difference in organic substances in comparison with “blank” (w/o NPK, w/o micronutrients) test, mean values for 4 hybrids

The maize hybrids KWS 381 and Karifols have a higher starch content, crude protein, and fat content. |In addition, the hybrid KWS 381 is characterized by the highest cellulose content among the studied samples (28.4–29.6% to DM). The application of macronutrients and micronutrients led to a relative increase in the content of starch, crude protein, and cellulose (Fig. 7).

In the research of Schittenhelm (2008), ash concentration was higher in late-ripening maize hybrids, and fat concentration increased until the end of the growing season in all hybrids. The differences in fat content in the whole plant between maize hybrids were more pronounced at later stages of development. A decrease in protein content was observed at late harvest dates.

At the same time, higher values of these indicators correspond to higher doses of applied macronutrients (NPK). In the case of cellulose, its average value increases with an increase in the dose of macronutrients and with the application of microfertilizers. Application of N_120_P_90_K_90_ in combination with YaraTera Tenso Cocktail seed treatment and YaraVita Kombiphos spray of maize plants provided the greatest increase in cellulose content, up to 7%, on average in the experiment.

Lamptey et al. (2018) established that application of N_300_, and to a lesser extent N_200_ decreases acid detergent fiber and neutral detergent fiber but increases crude protein compared to unfertilized plots.

Simultaneously, with an increase in cellulose content, there is an almost proportional decrease in fibre content under the influence of macronutrients and microfertilizers. There is also a noticeable increase in ash content in variants using macrofertilizers. However, no clear dependence of their content in DM was observed for the rest of the parameters with the application of macro- and micronutrients.

### Theoretical biogas yield estimation

The methane yield per hectare is predominantly influenced by the maize variety and the harvesting time (Amon et al. 2007; Zhao et al. 2016).

The general range of theoretically estimated CH_4_ specific yield based on the data on crude protein, crude fat, cellulose, and hemicellulose content in maize DM is 272.1 to 356.6 Nm^3^CH_4_ t^−1^VS. In terms of the fresh mass of maize, this amounts to 97.2–129.2 Nm^3^CH_4_ t^−1^FM. Therefore, the specific methane yield based on 1 ha of land area, with a yield of 13.8–18.6 tVS ha^−1^, will be within 3860–6630 Nm^3^CH_4_ ha^−1^.

**Fig. 8:**
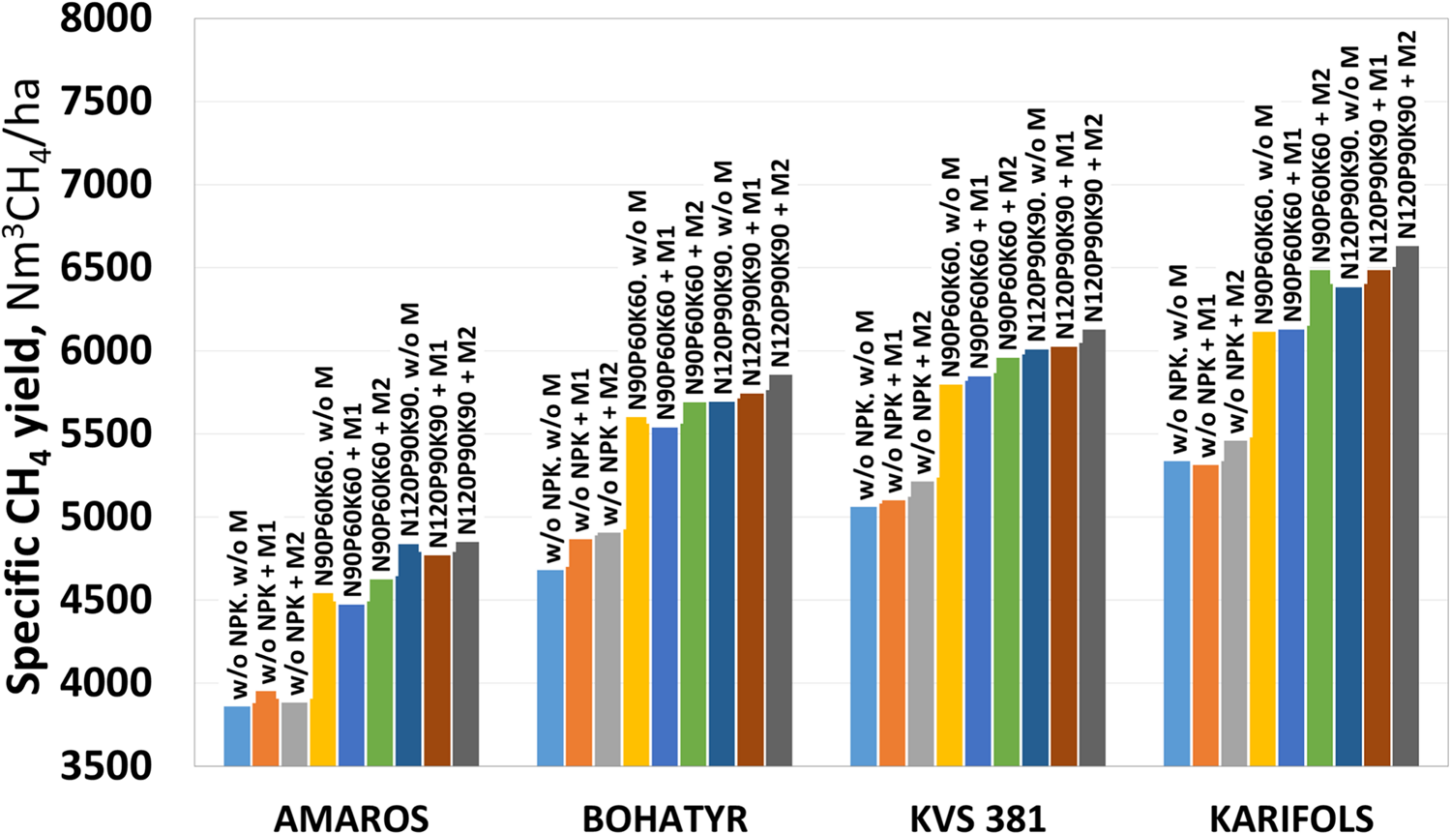
Methane yield per hectare from maize hybrids

The highest methane output potential was obtained for the Karifols maize hybrid — 5338–6630 Nm^3^CH_4_ ha^−1^, slightly less for the KWS 381 hybrid — 5062–6128 Nm^3^CH_4_ ha^−1^, for the Bohatyr hybrid — 4681–5856 Nm^3^CH_4_ ha^−1^ and the lowest potential was for the Amaros hybrid — 3861–4849 Nm^3^CH_4_ ha^−1^ (Fig. 8). In variants using macro- and micronutrients, the Karifols hybrid had a 36.6% higher CH_4_ output potential per 1 ha, compared to the Amaros hybrid.

This corroborates with the data obtained by Oechsner et al. (Oechsner et al. 2003), where methane yield per 1 ha is assumed to be consistent with an increasing maturity group of maize hybrids. The highest methane yield of 9370 Nm^3^ ha^−1^ was obtained for the hybrid with the latest maturity. Therefore, it appears that late-energy maize can take full advantage of the growing season.

According to Oslaj (2010), the late maturity hybrids of maize give higher methane yields — 7768.4 Nm^3^ ha^−1^ (FAO 400) and 7050.1 Nm^3^ ha^−1^ (FAO 500). Among the maize hybrids of maturity classes FAO 300–400, the hybrid PR38F70 gives the highest biomethane output per hectare (7646.2 Nm^3^ ha^−1^). Among the hybrids of FAO 400–500 maturity classes, the Pixxia hybrid achieved the highest biomethane output (9440.6 Nm^3^ ha^−1^). Among the hybrids of maturity classes FAO 500–600, the highest production of biomethane gives the Codistar hybrid (8562.7 Nm^3^ ha^−1^). Biomethane production varied with maize hybrids from 50 to 60%.

The application of macronutrients provides the most significant increase in CH_4_ output potential. Thus, the use of N_90_P_60_K_60_ allows to increase this indicator by 11.2–16.9%, compared to non-fertilized options. At the same time, increasing the dose of fertilizers from N_90_P_60_K_60_ to N_120_P_90_K_90_ allows to additionally increase the specific yield of CH_4_ by 1.7–6.5%.

The effect of micronutrients according to the F3 scheme is not significant when applied separately from macrofertilizers, and is practically absent in combination with the application of macronutrients (NPK). The potential of methane output in options without macronutrients use ranges from − 0.4 to + 4.0%, and when applying N_90_P_60_K_60_ from1.1 to + 0.8%, and in the case of N_120_P_90_K_90_ use from 1.4 to + 1.6%.

The use of micronutrients according to the F4 scheme can be more effective for the production of biomethane, which allows an increase in the potential of methane output in variants without macronutrients by + 0.8 to + 4.8% when applying N_90_P_60_K_60_ — from + 1.6 to + 6.1%, and when using N_120_P_90_K_90_ — from + 0.2 to + 3.9%. Thus, the use of macronutrients has a greater effect on the methane output potential than micronutrients.

### Energy and economic effects of applying different fertilizing schemes

The social question of use of agricultural land for growing bioenergy and not fodder crops asks for empirical values on the land demand per unit of energy produced, which should be as low as possible (Bauer et al. 2010; Zhao et al. 2016).

**Fig. 9:**
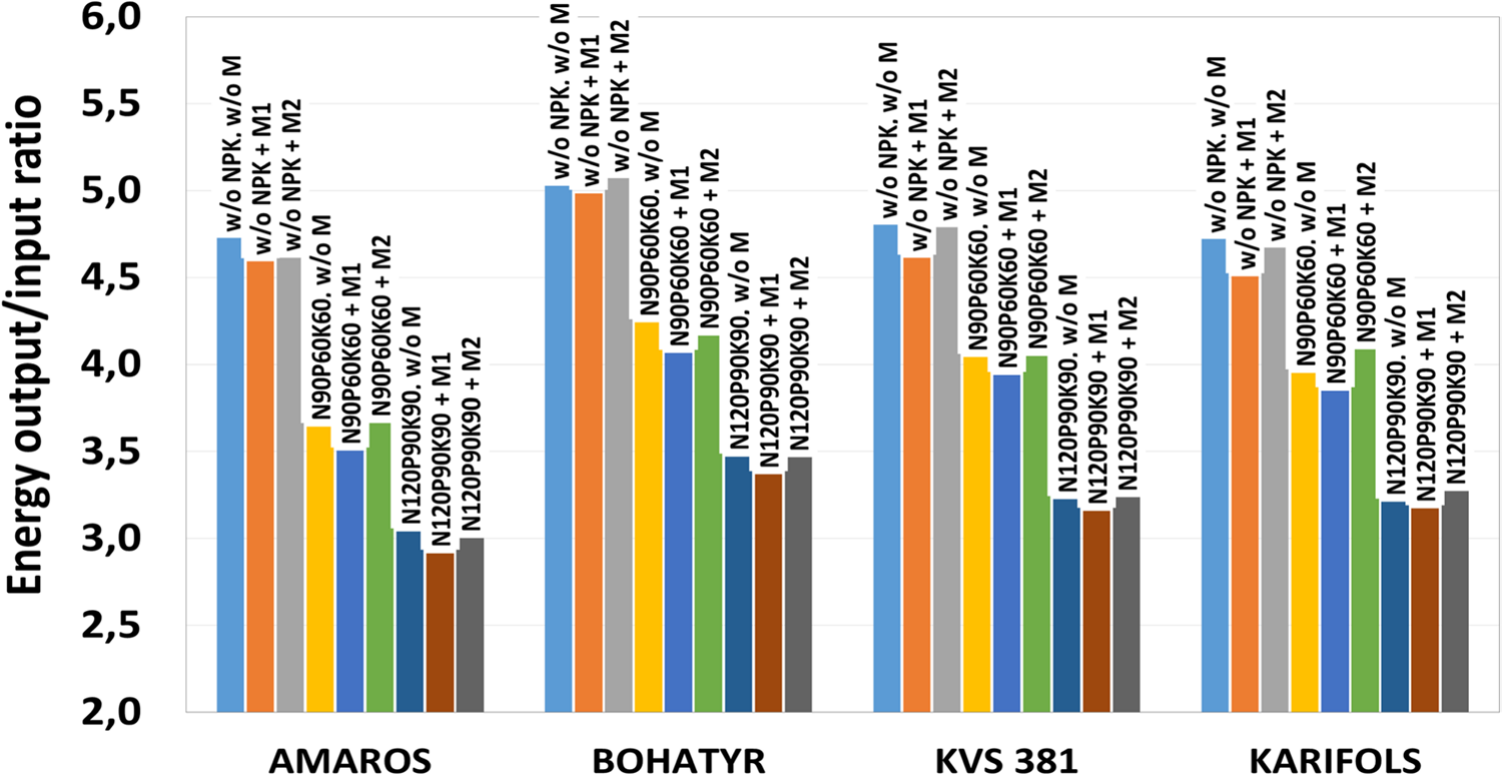
The energy efficiency of maize cultivation for biogas production (average for 2019–2021)

The ratio of the energy potential of biomethane from maize silage to the energy needed for its cultivation, collection and grinding amounts to 2.9–5.1 times. The energy demand (E_I_) for maize production is estimated at 25.7 to 46.1 GJ ha^−1^ or 0.8–1.4 GJ t^−1^FM of maize silage (Fig. 9).

According to Jankowski et al. (2020), mineral fertilizers are the important source of energy (72–73%) in the cultivation of maize, mainly due to the high energy value of mineral fertilizers (65–70%) and to a lesser extent due to the energy requirement during fertilization.

Due to the high energy demand for macronutrients and the increase in costs for their preparation and application, an increase in energy consumption (E_I_) was observed when using N_90_P_60_K_60_ by 34.8–53.0%, compared to the option without its use. Increasing the dose of macronutrients from N_90_P_60_K_60_ to N_120_P_90_K_90_ requires an additional 36.2–43.9% energy expenditure (E_I_). The application of micronutrients requires additional energy expenditure (E_I_) of 3.2–7.3%.

When applying macronutrients, a decrease in energy use efficiency (EUE) is observed. Thus, in the variant without the application of macronutrients, this indicator was 4.5–5.0, with N_90_P_60_К_60_ EUE decreased to 3.5–4.1, and with N_120_P_90_К_90_ EUE decreased to 2.9–3.5.

When using scheme F3 with microfertilizers, energy output from biogas (E_o_) increased by 1.3–4.4%, and energy consumption (EI) increased by 1.5–3.4%, compared to the option without their use. However, energy use efficiency (EUE) did not change practically. When using the F4 scheme of micronutrients, the energy production of biogas (E_o_) and energy consumption (EI) were 200.5–339.5 GJ ha^−1^ and 26.2–46.0 GJ ha^−1^, respectively. At the same time, the EUE was 3.0–4.8, which is 1.4–4.2% higher than the option without micronutrients.

It should be noted that most of the studied schemes of applying macro- and micronutrients make it possible to obtain from 0.4 to 16.4% of additional energy in biomethane compared to options without their application.

Our results corroborate with the research of other scientists. For example, in Poland, the energy output from maize silage ranged from 197 to 290 GJ ha^−1^, whereas the average energy output of sorghum silage was 61 GJ ha^−1^ lower. The energy efficiency ratio and the energy gain for maize were determined at 7.7–11.3 and 172–265 GJ ha^−1^, respectively (Jankowski et al. 2020). In Belgium, the energy output of maize biomass ranged from 319 to 363 GJ ha^−1^. In different production technologies maize biomass grown in Germany accumulated 300 − 368 GJ ha^−1^ of energy (Boehmel et al. 2008).

According to the experiment, operational expenditures for the cultivation and harvesting of maize were 208.1–605.8 euros ha^−1^ or 5.8–13.5 euros t^−1^ of silage. Conditionally, the gross income from selling biomethane from maize silage ranges from 307–757 euros/ha at a biomethane price of 200 euros per 1000 m^3^ to 3226–5364 euros ha^−1^ at a biomethane price of 1000 euros per 1000 m^3^.

**Fig. 10:**
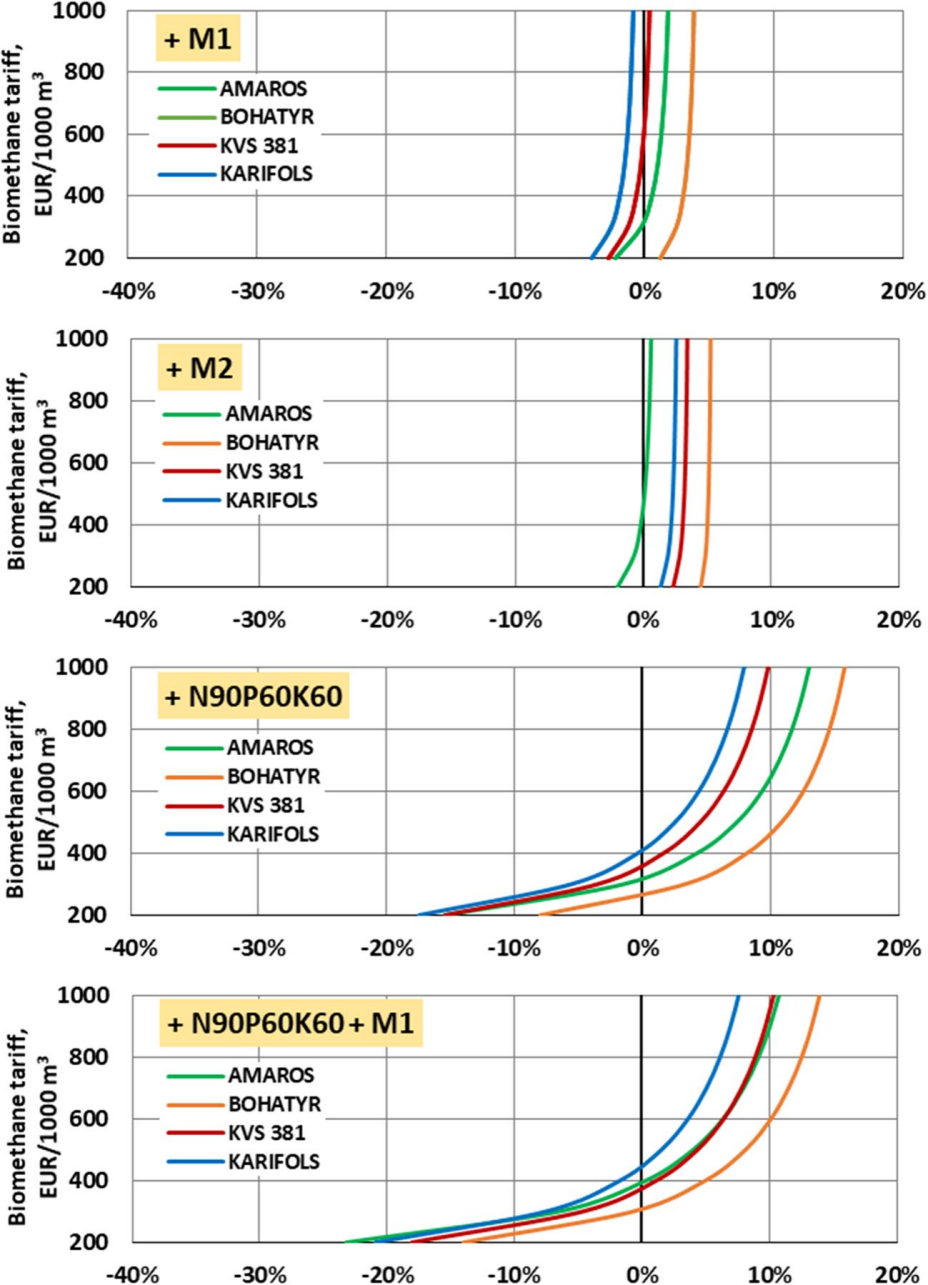

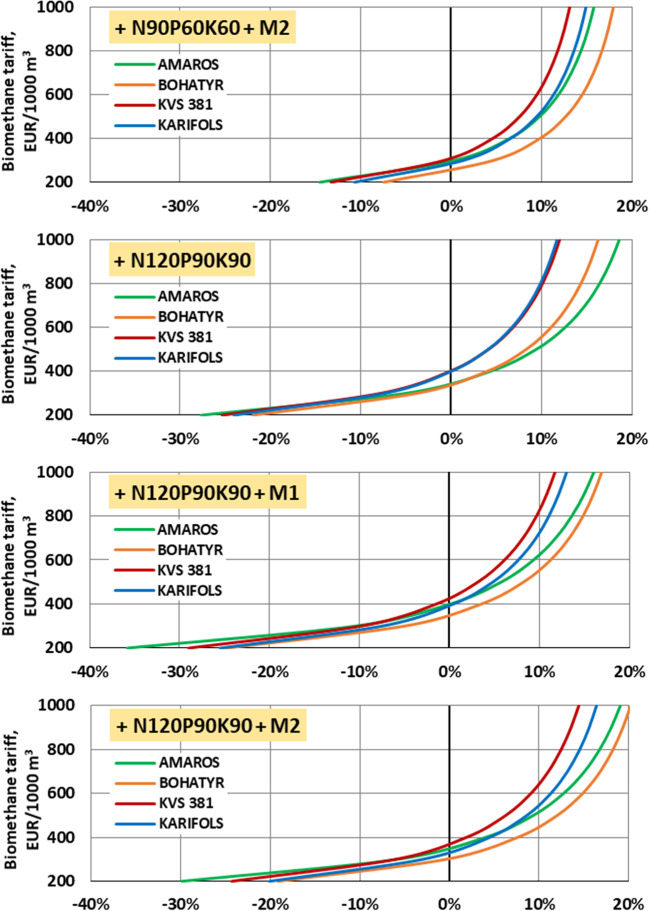
Relative difference in “net” profit “in the field” in comparison with “blank” test (w/o macronutrients, w/o micronutrients)

Figure 10 shows that from an economic point of view, at a cost of biomethane of 400 euros/1000 m^3^, the use of macronutrients and micronutrients to grow maize hybrids becomes profitable.

At a biomethane price of 1000 euros/1000 m^3^, applying only micronutrients according to the F3 scheme (Bogatyr hybrid) allows for increasing profitability by 3.8% only, and by 5.3%, according to the F4 scheme.

The application of N_90_P_60_K_60_ macronutrients allows for increasing profitability by 7.9–15.8%. Using macronutrients according to the F1 scheme and micronutrients according to F3 is economically impractical, as it leads to an increase in profitability by only 0.5% (KWS 381 hybrid). The application of micronutrients according to the F4 scheme is more economically feasible and increases the profitability of growing maize for biogas production by 2.2–7.0%.

Increasing the dose of macronutrients from N_90_P_60_K_60_ to N_120_P_90_K_90_ provides a slight economic effect, increasing profitability by 0.5–5.6%. Using macronutrients according to the F2 scheme and micronutrients according to the F3 scheme allows for increasing profitability of maize cultivation up to 1.3% (Carifols hybrid). When applying micronutrients according to the F4 scheme, the yield increases to 4.7% (Carifols hybrid).

Thus, for most of applied experimental schemes, the use of macronutrients and micronutrients is appropriate both from the energy point of view and from the economic point of view. The profitability of their use begins to appear at the price of biomethane of 300–400 euros/1000 m^3^. At the same time, the highest economic profit among the studied maize hybrids can be obtained from Bogatyr and Amaros maize hybrids when using N_120_P_90_K_90_ in combination with the use of micronutrients according to the F4 scheme. The KWS 381 maize hybrid provides the lowest economic efficiency. In Ukraine, it was determined that the use of macro and micronutrition when growing corn for silage to obtain biogas is economically and energetically appropriate. At the same time, according to various data (Černý et al. 2012; Houshyar et al. 2015; Dilip and Bao-Luo 2016), the use of fertilizers in the cultivation of corn for silage as a forage crop is not always ineffective. Therefore, more research is needed to study new combinations of macro and micronutrients when growing corn for silage.

## Conclusion

The use of macronutrients (NPK) leads to an increase in maize fresh mass productivity by 11.4–21.0%, DM productivity by 11.4–17.0%, and an increase in CH_4_ output potential by 11.2–30.9%, compared to options without their application. The use of micronutrients makes it possible to increase these indicators by 1.4–3.6%, 1.2–3.9%, and 1.8–3.6%, respectively. No clear dependence of the effect of macronutrients and micronutrients on the content of DM was found in the investigated maize hybrids. The application of macro- and micronutrients results in an increase in the content of starch, crude protein, ash and cellulose, and in a decrease in the fibre content. The use of macro- and micronutrients is expedient from both an energy and an economic point of view. The profitability of their use begins to appear at the price of biomethane of 300–400 euros/1000 m^3^. In further research, it is recommended to study new corn hybrids and different combinations of macro- and micronutrients.

## Supplementary Information

Below is the link to the electronic supplementary material.

Supplementary file1 (DOCX 276 KB)

## Data Availability

The datasets used and/or analysed during the current study are available from the corresponding author on reasonable request.
