# Supplementary material for: Influence of macronutrients and micronutrients on maize hybrids for biogas production
Source: Environ Sci Pollut Res Int. 2023 May 5;30(27):70022–38. doi: 10.1007/s11356-023-27235-3 (PMC10239385; doi:10.1007/s11356-023-27235-3)
Supplement: Supplementary file 1 — Supplementary file1 (DOCX 276 KB) [file 11356_2023_27235_MOESM1_ESM.docx]

**Supplementary Material**


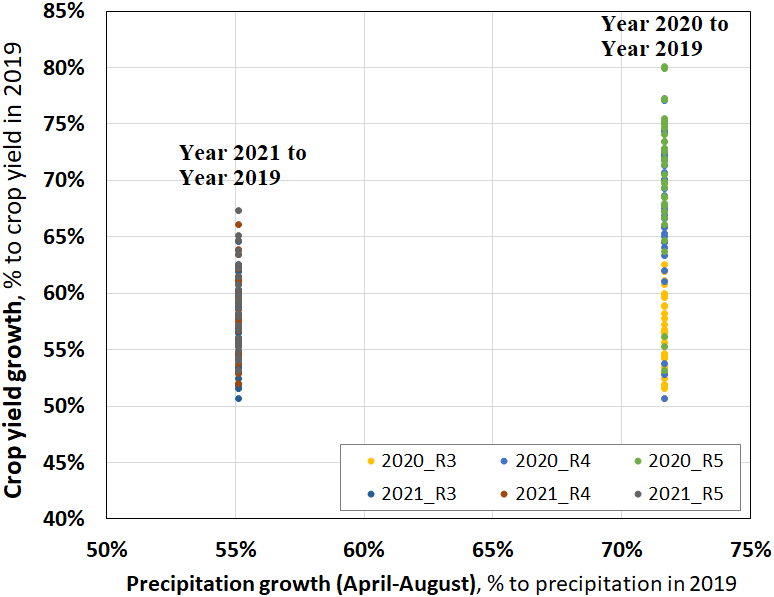


S.1 Relative increase of maize crop yield vs a relative increase of precipitation amount


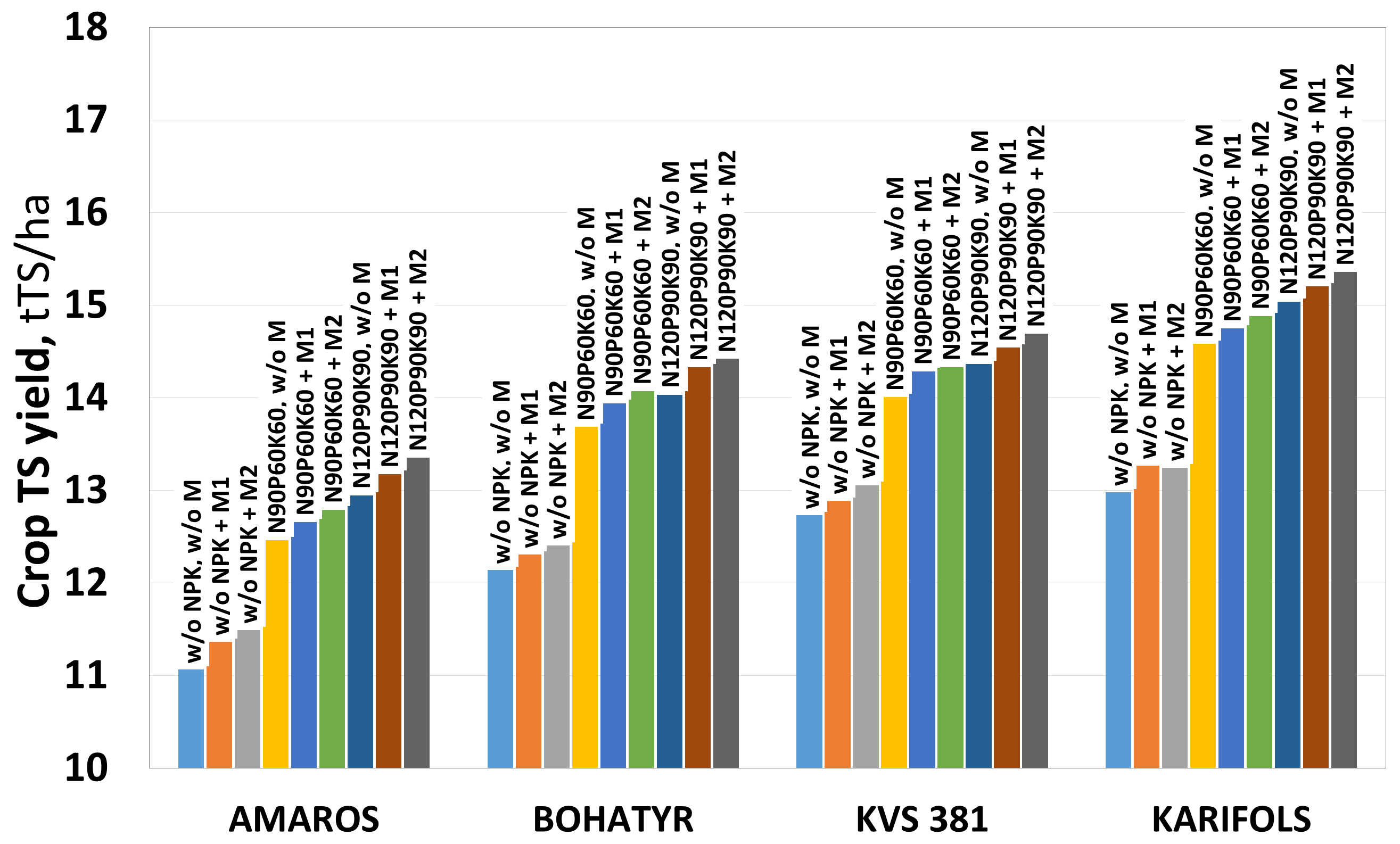


S. 2. Total dry matter yield per 1 ha for different maize hybrids (Kernel Milk Stage (R3)), average value for 2019-2021
